# The orthopedic characterization of *cfap298*^*tm304*^ mutants validate zebrafish to faithfully model human AIS

**DOI:** 10.1038/s41598-021-86856-1

**Published:** 2021-04-01

**Authors:** Laura Marie-Hardy, Yasmine Cantaut-Belarif, Raphaël Pietton, Lotfi Slimani, Hugues Pascal-Moussellard

**Affiliations:** 1 Orthopedic Surgery and Trauma Center, Pitié-Salpêtrière Teaching Hospital, 47 Boulevard de l’Hôpital, 75013 Paris, France; 2 Paris Brain Institute, ICM, Inserm U 1127, CNRS UMR 7225, Sorbonne Université, 75013 Paris, France; 3 EA 2496 Laboratory Orofacial Pathologies, Imaging and Biotherapies, Dental School University Paris Descartes Sorbonne Paris Cité, and Life Imaging Platform (PIV), Montrouge, France

**Keywords:** Disease genetics, Development of the nervous system, Genetics of the nervous system, Spine regulation and structure, Motility, Biomedical engineering, Medical research, Pathogenesis, Clinical genetics, Neurological disorders, Musculoskeletal abnormalities

## Abstract

Cerebrospinal fluid (CSF) circulation relies on the beating of motile cilia projecting in the lumen of the brain and spinal cord cavities Mutations in genes involved in cilia motility disturb cerebrospinal fluid circulation and result in scoliosis-like deformities of the spine in juvenile zebrafish. However, these defects in spine alignment have not been validated with clinical criteria used to diagnose adolescent idiopathic scoliosis (AIS). The aim of this study was to describe, using orthopaedic criteria the spinal deformities of a zebrafish mutant model of AIS targeting a gene involved in cilia polarity and motility, *cfap298*^*tm304*^. The zebrafish mutant line *cfap298*^*tm304*^, exhibiting alteration of CSF flow due to defective cilia motility, was raised to the juvenile stage. The analysis of mutant animals was based on micro-computed tomography (micro-CT), which was conducted in a QUANTUM FX CALIPER, with a 59 µm-30 mm protocol. 63% of the *cfap298*^*tm304*^ zebrafish analyzed presented a three-dimensional deformity of the spine, that was evolutive during the juvenile phase, more frequent in females, with a right convexity, a rotational component and involving at least one dislocation. We confirm here that *cfap298*^*tm304*^ scoliotic individuals display a typical AIS phenotype, with orthopedic criteria mirroring patient’s diagnosis.

## Introduction

### Zebrafish is a frequently used animal model in genetic and could be used to investigate AIS

In human, adolescent idiopathic scoliosis (AIS) is by far the most common form of non-congenital scoliosis seen in practice, occurring in the absence of associated pathologies^1–3^. It is the most frequent musculoskeletal disorder occurring in 1–3% of the population, arising in childhood and worsening before the peak of growth during adolescence. If the frequency of AIS is higher in female patients (80%), no major differences in the curves pattern has been demonstrated yet^4,5^. If untreated, AIS can lead to chronic low back pain, pulmonary restrictive syndrome and severe disabilities along life^2,6–10^. While bracing is an efficient way to contain the progression of spinal curves, surgical correction may be needed to correct severe and resistant curves. Thus, a clear understanding of patients’ physiopathology is essential to appreciate the clinical behavior and may help to prevent the disease by identifying the progression factors and then individually adapt treatments.

Among genetic model organisms helping to understand AIS pathogenicity and etiology, zebrafish has recently emerged as an advantageous animal model^11–13^. A biomechanical study in finite elements recently showed that the zebrafish spine is a relevant model of human spine deformation^14^. While scoliosis occured rarely in quadrupedal animals, the longitudinal shape of zebrafish coupled with their ability to experience spinal loads in water make them naturally sensitive to three-dimensional spine deformities, which they even naturally develop with elderliness, highly similarly to humans^15,16^. Recent studies have demonstrated a link between spinal curves and cilia motility involved in cerebrospinal fluid (CSF) circulation^11–13,17,18^, as mutants defective in cilia motility develop AIS phenotypes. A cilium-linked physiopathology in AIS patients is highly suspected, as mutations related to cilium has been found in AIS cohorts (POC5, PAX1) and due to the correlation between CSF-flow and AIS illustrated by the higher prevalence of scoliosis in AIS patients^19–22^. Cfap298 is a gene involved in cilium dysfunction, that has been investigated on a Zebrafish model, concluding to spine deformities mimicking AIS, as well as ptk7 mutants for example^11–13,23^. However, in these studies, the orthopedic characterizations of spinal deformities are sometimes lacking analysis through the eyes of clinicians used to analyze spine deformities, scoliosis, among others.

Indeed, although zebrafish are susceptible to develop spinal curvatures naturally or upon targeted gene mutations, it is not clear whether these AIS animal models faithfully recapitulates the attributes of human pathologies of the spine. Here, using micro-computed tomography and clinical criteria, we characterized spine deformities developed in the zebrafish mutant line *cfap298*^*tm304*^ that affects cilia motility and polarity to confirm the link between these deformities and AIS^12,24,25^.

## Methods

### A genetically modified zebrafish for cilium related gene and exhibiting spine deformities was investigating in micro-CT

#### Animal husbandry

All procedures were performed on juvenile and adult zebrafish in accordance with the European Communities Council Directive (2010/63/EU) and French law (87/848) and approved by the Paris Brain Institute (Institut du Cerveau). An approval agreement was obtained from the French ethic committee for experimentation on juvenile and adult zebrafish (APAFIS agreement number 2018071217081175). All experiments were performed on *Danio rerio* of AB, Tüpfel long fin (TL) and nacre background. The study was carried out in compliance with the ARRIVE guidelines. Animals were obtained from a natural mating and were raised under a 14/10 light/dark cycle.

This study did not involve human subjects. All methods were carried out in accordance with relevant guidelines and regulations.

#### Induction of cilia motility defects and spine deformities

27 sibling animals were obtained from the natural mating of a *cfap*^*tm304/*+^ male and a *cfap298*^*tm304/tm304*^ female^24,25^, resulting in the progeny in 50% of homozygous mutant animals (n = 17 animals analyzed) and 50% of heterozygous siblings (n = 10 animals analyzed) . As the *cfap298*^*tm304*^ mutation is thermosensitive, the induction of cilia motility defects was based on a temperature shift as described in a previous work^12^. Animals were first raised at 25 °C until 6 days post-fertilization (dpf) to allow normal embryonic development and then switched at 30 °C from 18 to 23 dpf as described in a previous work^12^. Homozygous mutant animals subjected to this restrictive temperature (30 °C) developed defects in spine alignment as previously reported. Heterozygous animals were subjected to the same temperature shift and used as control siblings.

#### Scannographic analysis

Juvenile zebrafish were imaged at 8 and 12 weeks old under general anesthesia using 0.02% MS-222 (Sigma). Micro-Computed Tomography (micro-CT) was performed using a QUANTUM FX CALIPER, with a 59 µm – 30 mm protocol. The DICOM images were analyzed with RADIANT DICOM VIEWER 5.0.0 software. All scannographic analyses were performed on three-dimensional reconstructions, oriented in sagittal and coronal planes as described in^26^ and were performed by an orthopedic surgeon to avoid bias. The thoracic and lumbar segments of the zebrafish’s spines were defined according to the sagittal alignment: zebrafishes present one long thoracic kyphosis followed by a lumbar lordosis. The classical criteria for scoliosis characterization taken into account in this study were the number of spine curves, the side of the main curve, the Cobb angles, the number of dislocations, the Lenke classification^27^, apical vertebral rotation (AVR) and apices of the curves. The Lenke classification was applied to the zebrafish’s spines considering the main curves and the contra-curves (I: main thoracic, II: double thoracic, III: double major, IV: triple major, V: thoraco-lumbar/lumbar and VI: thoraco-lumbar/lumbar/main thoracic), without the sagittal modifier rules. Dislocations were analyzed in 3D reconstruction, as known to be more precise than 2D measurements and defined by an AVR > 10° associated to a lateral listhesis on the frontal plane between two adjacent vertebras^28^. They were measured from frontal reconstructions as described in Marie-Hardy et al.^26^.

#### Statistical analysis

All values are represented as histogram distributions or mean ± SEM (stated for each in the figure legend). Differences were analyzed with two-tailed-t-tests. Significance was set at 0.05. Statistical details related to sample size in each group and p-values are reported in the figures and figure legends. Asterisks denote the statistical significance: *p < 0.05; **p < 0.01; ***p < 0.001; ns, *p* > 0.05.

## Results

### 63% of the zebrafish cohort exhibits a three-dimensional spinal deformity of the spine with right convexity, similar to AIS

*cfap298*^*tm304*^ zebrafish carries a temperature sensitive mutation in the cilia motility gene *cfap298*, where cilia beating can be inactivated in a temporally controlled manner to alter CSF flow in brain and spinal cord cavities^12^. Shifting the environmental temperature of the animals from permissive (25 °C) to restrictive (30 °C) results leads to the development of three-dimensional spinal deformity of the spine during growth^12^. While the zebrafish appears to be an effective model for exploiting the pathogenicity hypotheses underlying the development of AIS, its usefulness for orthopaedic research lacks validation with the clinical criteria used for the diagnosis of human patients.

Here, we took advantage of the *cfap298*^*tm304*^ mutation to characterize spinal deformities on the basis of orthopedic criteria and to fully validate this AIS model. We performed micro-CT imaging on a cohort of 17 homozygous mutant animals (*cfap*^*tm304/tm304*^) and 10 heterozygous siblings (*cfap298*^*tm304/*+^), which were subjected to a temperature shift to allow the development of spinal misalignment in the homozygous mutant experimental group (see “Methods” for details).

**Figure 1:**
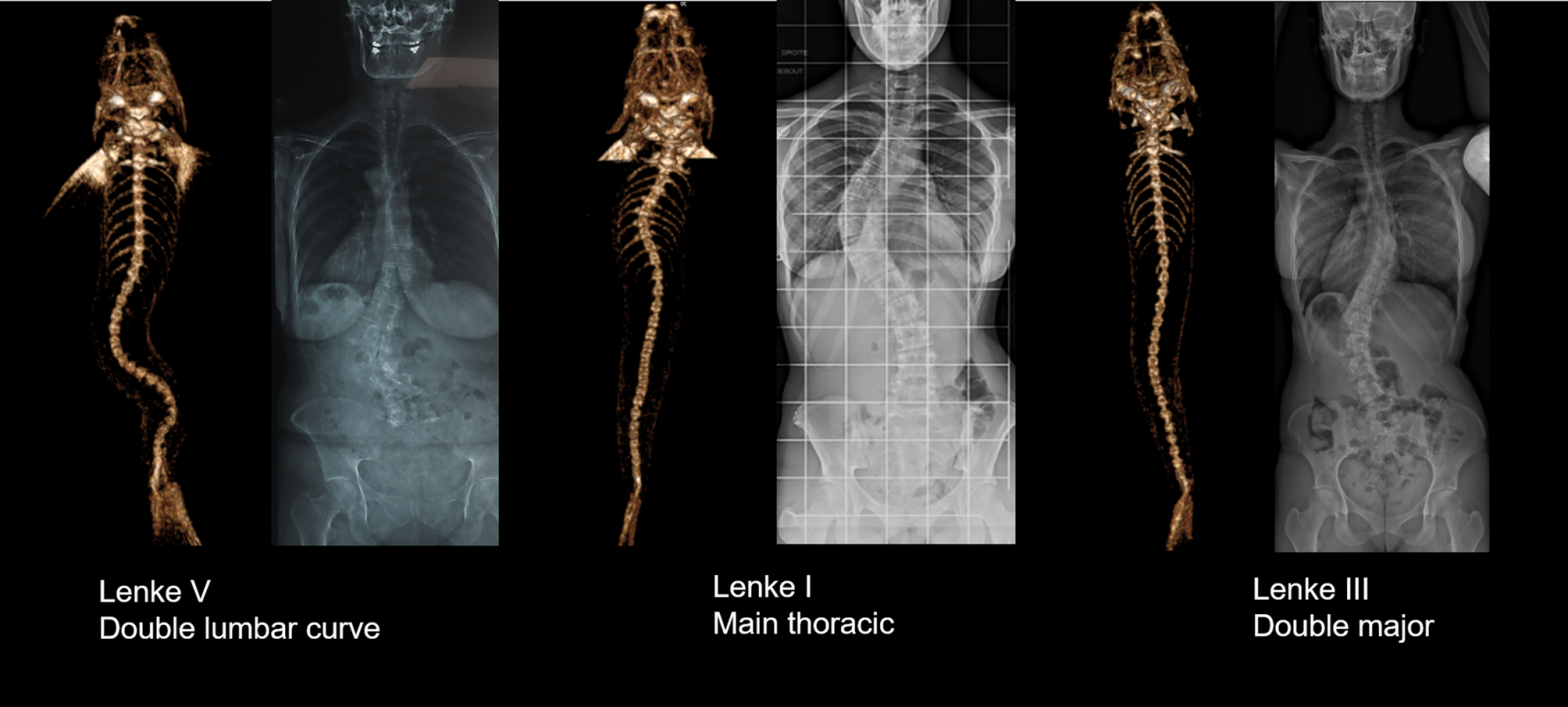
Three examples of frontal 3D-reconstructions of zebrafish’s spine deformities, classified according to Lenke and compared to corresponding human scoliosis. Left: Reconstructions from RaDiant Dicom Viewer 5.0.0 software (https://www.radiantviewer.com/); Right: Clinical plain radiographs (personal collection of LMH).

**Figure 2:**
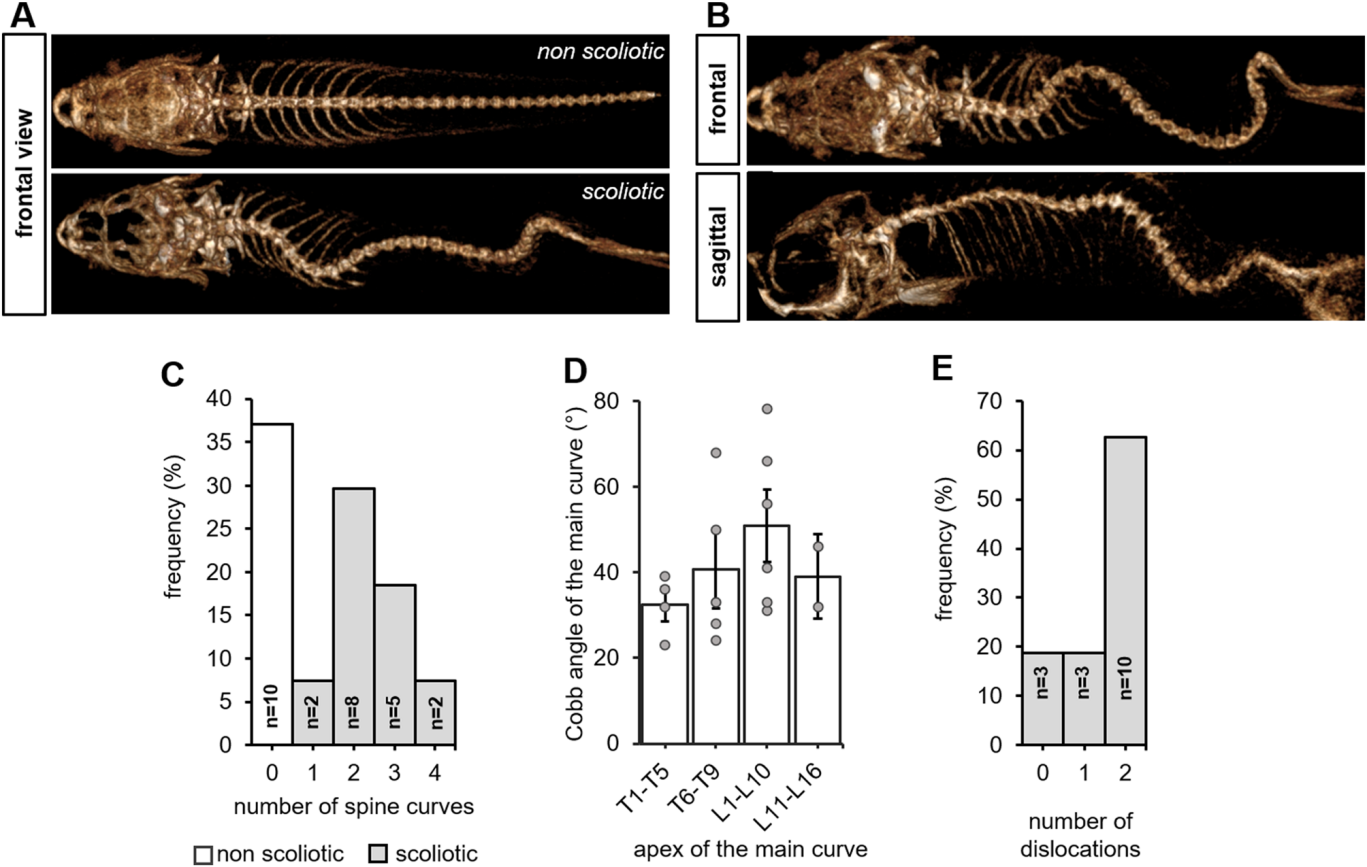
Juvenile cfap298^*tm304/tm304*^ zebrafish mutants develop an evolutive thoraco-lumbar curvature of the spine. (**A**) Frontal views of scannographic reconstructions of 8-week-old non-scoliotic (top) and *cfap298*^*tm304*^ scoliotic sibling (bottom). (**B**) Frontal and sagittal view of the same *cfap298*^*tm304*^ scoliotic fish after scannographic reconstruction showing a 3-dimensional torsion of the spine reminiscent of AIS. (**C**) Distribution of the frequency of the number of spine curves observed in the sagittal plane of scoliotic (grey) and non-scoliotic (white) animals. The population analyzed was raised from a cross of *cfap298*^*tm304/*+^ with *cfap298*^*tm304/tm304*^. Most of *cfap298* scoliotic animals display double or triple curves in the frontal plane. (**D**) Distribution of the Cobb angle of the main curve in *cfap298* scoliotic fish depending on the localization of the apex of the main curve. Bars represent the average Cobb angles ± SEM. Each point represents a single fish. Most of the main curve apices are located between T6 and L10 (11 out of 17 fish) (**E**) Histogram showing the distribution of the number of dislocations observed in the scoliotic population (frequency, %).

Radiological confirmation of a suspected scoliosis relies on the observation of a three-dimensional and rotational spinal deformity characterized by a thoracic curvature in the frontal plane with a Cobb angle greater than 10 degrees on frontal radiographs^1,3^. Here, we observed that 63% of the *cfap298*^*tm304*^ population analyzed (n = 17 out of 27 animals) had three-dimensional spinal deformity in the frontal and sagittal planes (Fig. 2A, B), with curves exceeding 10 degrees in the frontal plane (42.1° ± 4.1°, [min–max: 23°–78°]). In the scoliotic population analyzed, we observed that most of the animals had double or triple curves (n = 13 out of 17 scoliotic fishes) (Fig. 2C). If classified as human adolescent idiopathic scoliosis, 29% of the curves were Lenke 3, 29% Lenke 4, 18% Lenke 5, 6% Lenke 1 and 6 or 1 and 2 animals were unclassifiable according to Lenke classification^27^. The Fig. 1 displays some example of the Lenke classification applied to Zebrafish spine.

To fully describe the curve pattern of scoliotic animals, we measured the amplitude of the main curve at 8 weeks old, and observed it was larger when the apex was located in the thoraco-lumbar junction (Fig. 2D), reflecting the anatomical location of the main curve observed in the patients. Moreover, the magnitude of the main curve for the 17 scoliotic zebrafishes was 42.1° ± 4.1°, [min–max: 23°–78°], while we observed a mean angle of 36.5° ± 6.7° [min–max: 13°–77°] for the first minor curve and 27.9 ± 4.8°, [min–max: 16°–43°] for the second compensatory curve.

Spinal deformities associated with AIS in human pathology are also characterized by a progression of the curve severity over time, most notably during the period preceding the pubertal growth spurt^29^. Thus, we analyzed the evolution of the curve severity on scoliotic *cfap298*^*tm304*^ animals between 8 and 12 weeks of age. The mean length of the zebrafishes (all cohort) at 8 weeks old was 15.3 mm ± 1.86; [11.7;18.9 mm] and 19.8 mm ± 3.37 [12.4;25.5 mm] at 12 weeks old. We observed that the mean Cobb angle of the scoliotic *cfap298*^*tm304*^ zebrafish increased by 5.5° ± 7.3° (mean ± SEM) during this period, showing the progressive development of spine torsion. The mean Cobb angle for the second curve at 12 weeks was 47.6° ± 13° [29; 82°]. Moreover, the mean apical vertebral rotation for the main curve was 31° ± 13° [min–max: 20°–69°], reflecting its three-dimensional shape. The analysis of the curve patterns at 12 weeks also showed that spine curves remained mainly thoracic or thoraco-lumbar with double or triple curves and that 82% of the scoliotic juvenile fishes presented at least one dislocation (Fig. 2E). The dislocations were located at the thoraco-lumbar junction or at the lumbar spine for 79% of the animals.

**Figure 3:**
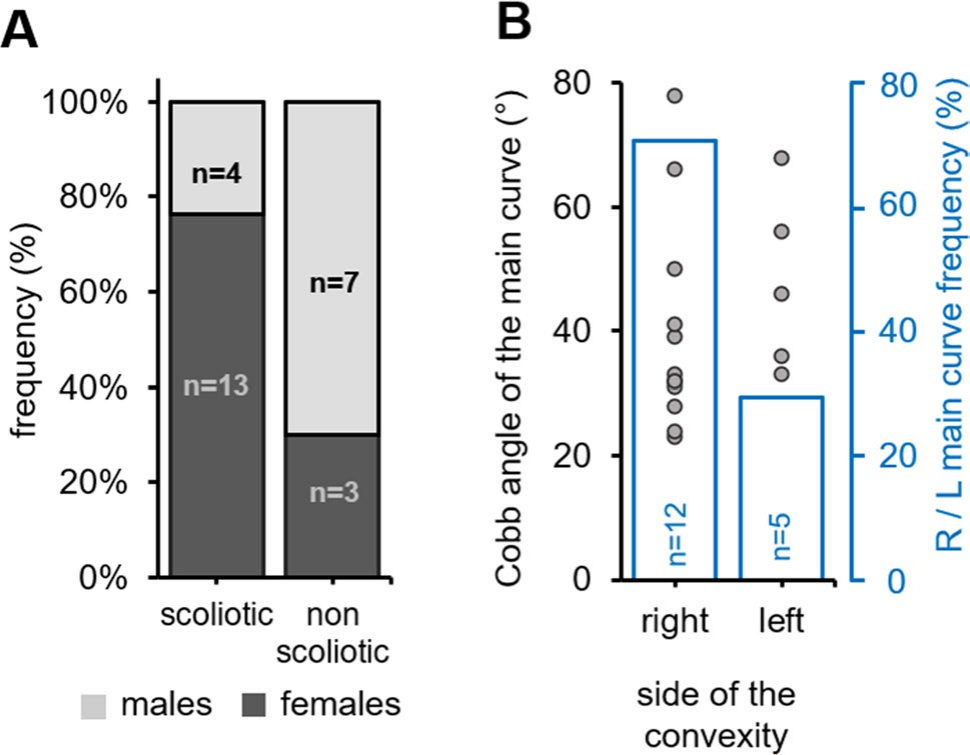
*cfap298*^*tm304*^ mutants exhibit a sexual and a right convexity bias for scoliosis development. (**A**) Stacked histogram showing the distribution of scoliotic and non-scoliotic phenotypes (frequency, %) in the analyzed cohort according to the gender (males: light grey, females: dark grey). Spinal curves were more prevalent in females (dark grey). (**B**) Distribution of the Cobb angle of the main curve in *cfap298*^*tm304*^ scoliotic fish according to the side of the convexity in the frontal plane (right versus left, grey points; each point represents a single fish). The frequency of the convexity of the main curve is represented in blue. Note that most of the curves are biased to the right side (12 out of 17 fishes).

Another feature of AIS is its sexual dimorphism. While female are a least more likely to develop scoliosis by a ratio of two^30,31^ and most severe curves are ten times most prevalent in females than males^32^ and the risk of progressive deformity requiring surgical treatment is five times higher in girls than in boys^33^. Thus, we compared the frequency of scoliosis occurrence in male and female siblings obtained from the cross of *cfap298*^*tm304/*+^ and *cfap*^*tm304/tm304*^ parents (Fig. 3A). At 8 weeks old, 76% (n = 13 out of 17 animals) of scoliotic fish were females, compared to 30% in the non-scoliotic population (n = 3 out of 10), suggesting a female bias in the penetrance of scoliotic curves. Moreover, only 4/13 (31%) of the male fishes developed scoliosis, compared to 13/14 (93%) of the females, that difference being statistically significative according to Fischer’s test (*p* = *0.001*). Although curve patterns may vary in AIS patients, right-sided thoracic deformities are by far the most common^34^. Figure 3B shows the distribution of the convexity of the main thoracic curve in *cfap298*^*tm304*^ scoliotic animals, which was located to the right in 71% of the scoliotic fish. Overall, these results showed that the cilia-defective *cfap298*^*tm304*^ mutant displayed characteristics of spinal curvature defects observed in human AIS patients.

## Conclusions

### The ***cfap298***^***tm304***^ mutant seems to be after orthopaedic analysis of the curves a relevant model of AIS

The scannographic analysis of *cfap298*^*tm304*^ mutants at two different ages (8 and 12 weeks) conducted in this study is based on orthopedic criteria. It indicates the presence of dislocations for 82% of the cohort as well as a three-dimensional deformity of the spine with a frontal Cobb angle above 10° for 63% of the scoliotic animals analyzed. The presence of an apical rotation is a clue element to define scoliosis rather than other spine deformities and was found at 31° ± 13° in this cohort, which is a strong argument for scoliosis. As previously described^12^, spine deformities are seen precociously, at 8 weeks old in *cfap298*^*tm304*^ mutants^35^. Consistently with previously reported AIS phenotype, we characterize here the evolutive severity of spinal deformities associated with scoliosis between 8 and 12 weeks. An increase of 5.5° was observed in four weeks, for an average Cobb angle of the main curve reaching 47.6° ± 13°.The predominance of a right thoracic convexity more frequent in female is also coherent with AIS^36,37^. Thus, our orthopedic analysis of spinal deformities confirms the relevance of the mutant *cfap298*^*tm304*^as a model for human AIS^12,38,39^.

One limitation of this study is of course the number of animals in the cohort, that render some statistics regarding male–female dimorphism and analysis of the curves weak. However, the analysis in an orthopaedic fashion does provide the clue elements to firmly link this phenotype to AIS.

How can we go further on the link between the *cfap298*^*tm304*^ mutation and the pathogenicity of AIS? The *cfap298*^*tm304*^ mutation affects a cytoplasmic protein that is expressed at the base of cilia, little sensory and motile organelles protruding from the surface of specialized cells in the organism^24^. Cilia dysfunction may be clinically linked to AIS, since variants in ciliary genes, especially involved in cellular mechanotransduction (LBX1: spinal cord differentiation, somatosensory signal transduction, POC5: centrin and inversin interaction in the centrioles, GRP126: axons myelinization), were found in AIS patients^20,21,40,41^. Recent literature focusing on AIS etiology also highlighted a link with elongated osteoblasts cilium, related to several transduction genetic defects have been found in AIS patients, suggesting a complex molecular and cellular cilium involvement in scoliosis^17,42^. Impairments in the inner ear system (lateral semi-circular canal asymmetry and vestibular canals morphology) possibly linked to cilia defects have also been identified in AIS patients and suggest the involvement of the vestibular system to keep the spine properly aligned^43–45^. no clear mechanism has emerged from these tissue-specific candidates.

On contrary, cilia beating in the brain and spinal cord cavities have been thoroughly explored and investigated recently in zebrafish. A link between CSF flow and AIS seems particularly relevant due to the frequent association of type I Chiari malformation with scoliosis and the possible regression of the spinal curves observed in these patients after a sub-occipital decompression^19^. The initial finding that disturbing CSF flow generates three-dimensional spine deformities placed cilia and CSF as essential players for keeping the spine straight during the juvenile period of growth. This explanation had recently been clarified and deepened by the generation of zebrafish mutants targeting the SCO-spondin protein forming the Reissner fiber. While cilia beating is necessary to form this acellular thread bathing in CSF^46^, zebrafish mutants devoid of this fiber have recently been shown to develop AIS-like spinal curves^47,48^, possibly with an involvement of the signaling pathway of Urotensin-related peptides^39,49^. These early forays into the genetics of AIS in zebrafish together with our orthopedic characterization of the *cfap298*^*tm304*^ mutant bolsters now the use of this model to find novel mechanisms regulating spine alignment.
