# Author Correction: The orthopedic characterization of *cfap298*^*tm304*^ mutants validate zebrafish to faithfully model human AIS

**DOI:** 10.1038/s41598-021-95273-3

**Published:** 2021-08-02

**Authors:** Laura Marie-Hardy, Yasmine Cantaut-Belarif, Raphaël Pietton, Lotfi Slimani, Hugues Pascal-Moussellard

**Affiliations:** 1 Orthopedic Surgery and Trauma Center, Pitié-Salpêtrière Teaching Hospital, 47 Boulevard de l’Hôpital, 75013 Paris, France; 2 Paris Brain Institute, ICM, Inserm U 1127, CNRS UMR 7225, Sorbonne Université, 75013 Paris, France; 3 EA 2496 Laboratory Orofacial Pathologies, Imaging and Biotherapies, Dental School University Paris Descartes Sorbonne Paris Cité, and Life Imaging Platform (PIV), Montrouge, France

Correction to: *Scientific Reports* https://doi.org/10.1038/s41598-021-86856-1, published online 01 April 2021

The original version of this Article contained errors in the spelling of the authors Laura Marie-Hardy, Yasmine Cantaut-Belarif, Raphaël Pietton, Lotfi Slimani and Hugues Pascal-Moussellard, which were incorrectly given as Marie-Hardy Laura, Cantaut-Belarif Yasmine, Pietton Raphaël, Slimani Lotfi and Pascal-Moussellard Hugues respectively.

The original Article has been corrected.

